# Treating intrahepatic cholangiocarcinoma with pemigatinib: two case reports of Nordic patients

**DOI:** 10.2340/1651-226X.2025.42073

**Published:** 2025-04-15

**Authors:** Vesa T. Väliaho, Iben Spanggaard

**Affiliations:** a Department of Oncology, Turku University Hospital and University of Turku, Turku, Finland; b Department of Oncology, Rigshospitalet Copenhagen University Hospital, Copenhagen, Denmark

**Keywords:** Cholangiocarcinoma, CCA, iCCA, *FGFR2*, pemigatinib, Finland, Denmark

## Abstract

**Background:**

Cholangiocarcinoma (CCA) is a diverse group of aggressive liver tumors with up to 20% being intrahepatic CCA (iCCA). Up to 15% of patients with iCCA have fibroblast growth factor receptor 2 (*FGFR2*) fusions or rearrangements. Here we evaluated iCCA treatment with pemigatinib, a selective inhibitor of *FGFR1–3*, in two patients from Denmark and Finland.

**Patients:**

We identified a total of two Nordic patients with iCCA in our clinics, who received first-line cisplatin/gemcitabine before initiating pemigatinib.

**Results:**

Case 1 was a 34-year-old woman with aggressive, metastatic iCCA upon presentation, who progressed on cisplatin/gemcitabine. Pemigatinib was initiated after *FGFR2* fusion detection by genomic testing. She had a partial response after three cycles (9 weeks) of pemigatinib but experienced disease progression after three more pemigatinib cycles. Adverse events were primarily managed by supportive care and dose reduction, except hyperphosphatemia, which was complicated by food allergies and required medication. She received subsequent chemotherapy but deteriorated rapidly and died 1 month later.

Case 2 was an 81-year-old man with unresectable iCCA who achieved stable disease with first-line chemotherapy. He switched to pemigatinib after *FGFR2* fusion detection by next-generation sequencing. The tumor shrank by 20% after three pemigatinib cycles and completely calcified with continued treatment. Adverse events were managed by two dose adjustments. Treatment has continued for 57 months and is ongoing.

**Interpretation:**

CCA is an aggressive disease that requires early molecular testing of abundant biopsy tissue so not to delay second-line therapies, such as pemigatinib. Variability in treatment outcomes is expected.

## Introduction

Cholangiocarcinoma (CCA) is a diverse group of aggressive tumors arising from the biliary epithelium [1], with ≈10%–20% being intrahepatic CCA (iCCA) [1–3]. Molecular profiling of CCA tumors revealed high genomic heterogeneity, with 40%–50% of patients harboring ≥1 clinically actionable genomic alteration, including alterations in the fibroblast growth factor receptor 2 (*FGFR2*) gene [1, 4]. *FGFR2* fusions and rearrangements are oncogenic drivers present in 10%–15% of patients with iCCA [5, 6]. Constitutional activation of *FGFR2* rearrangements in the absence of ligand binding promotes CCA tumor development through the RAS-ERK signaling pathway [5].

Efficacy and safety of oral pemigatinib, a potent, selective inhibitor of *FGFR1–3* [7], were demonstrated in the open-label, phase 2 FIGHT-202 study [8]. Objective response rate (95% CI) in patients with previously treated advanced CCA with *FGFR2* fusions/rearrangements was 37% (28%–47%). Hyperphospha-temia was the most common treatment-emergent adverse event (TEAE) in FIGHT-202 but did not result in treatment discontinuation. Pemigatinib was approved in 2021 in Europe for treatment of adults with locally advanced or metastatic CCA with an *FGFR2* fusion or rearrangement that progressed after ≥1 prior line of systemic therapy [7].

Here we present two cases of patients with iCCA treated with pemigatinib from Finland and Denmark from cases treated within our clinics since the approval of pemigatinib for iCCA in 2021. The first illustrates response to pemigatinib in a patient with aggressive cancer; the second presents an elderly patient with long-term response to pemigatinib.

## Results

### Case 1: Patient history, diagnosis, disease progression, and pemigatinib treatment

**Figure 1:**
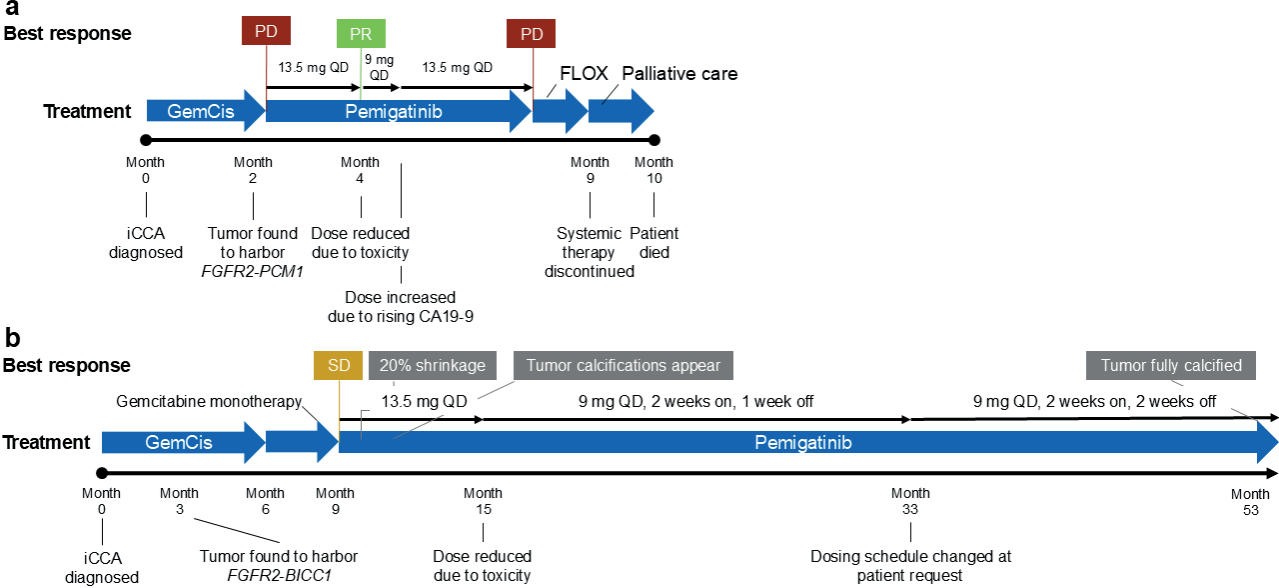
Summary of key events in the treatment of patients with iCCA. (a) Case 1 with aggressive, chemotherapy-resistant iCCA and (b) Case 2 of a patient with iCCA receiving long-term pemigatinib. CA19-9: carbohydrate antigen 19-9; FGFR2: fibroblast growth factor receptor 2; FLOX: oxaliplatin and 5-fluorouracil chemotherapy; GemCis: gemcitabine + cisplatin; iCCA: intrahepatic cholangiocarcinoma; PD: progressive disease; PR: partial response; QD: once daily; SD: stable disease.

**Figure 2:**
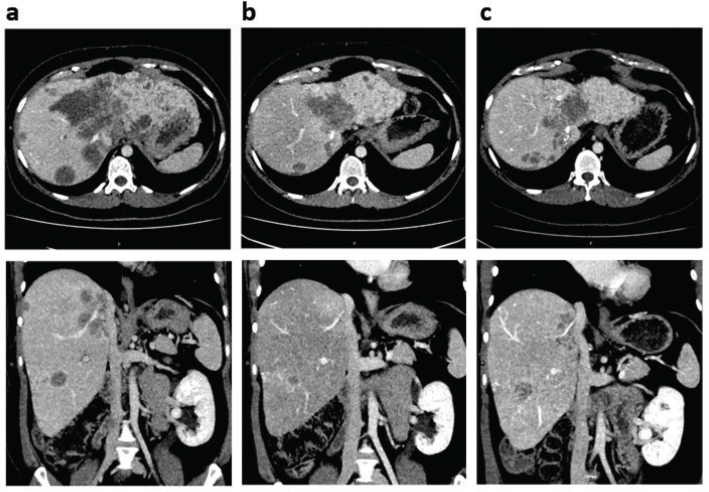
Partial response followed by disease progression on pemigatinib in the patient with aggressive, chemotherapy-resistant CCA. CT images show (a) disease progression after three cycles of GemCis chemotherapy, (b) partial response after three cycles of pemigatinib, and (c) disease progression after seven total cycles of pemigatinib. Axial and coronal views are shown on the top and bottom rows, respectively. CCA: cholangiocarcinoma; CT: computed tomography; GemCis: gemcitabine + cisplatin.

The patient was a 34-year-old woman at CCA diagnosis. Her relevant medical history included irritable bowel syndrome, severe atopic dermatitis, and extensive allergies, including to several foods, with no family history of gastrointestinal cancer. At a clinic visit 7 months before receiving her CCA diagnosis, she showed modestly elevated liver transaminases without abnormal abdominal ultrasound findings. Seven months later, she sought emergency medical care for upper abdominal pain, decreased appetite, and vomiting. Computed tomography (CT) imaging revealed a 14-cm malignant liver tumor, later biopsy-confirmed as iCCA. Multiple pulmonary metastases and suspicious lymph nodes were observed. Carbohydrate antigen 19-9 (CA19-9) was as high as 407 U/mL (normal range: <27 U/mL). The patient received three cycles of first-line cisplatin and gemcitabine (Figure 1a). Therapy was well tolerated; however, she experienced ongoing nausea and pain attributed to the tumor. After treatment with standard-of-care therapy, disease progression in primary tumor and pulmonary metastases was evident by CT scan (Figure 2a).

Genomic testing of biopsy tissue with the next-generation sequencing (NGS) FusionPlex Comprehensive Thyroid and Lung kit (ArcherDx, Boulder, CO, USA) was performed after initiation of first-line treatment, and *FGFR2-PCM1* fusion was detected. After disease progression, pemigatinib was initiated at 13.5 mg once daily (QD) on an intermittent dosing schedule (2 weeks on followed by 1 week off, per labeled indication). Treatment continued for three cycles, and the patient experienced a partial response (PR). Consistent shrinking of liver lesions was noted on CT, whereas pulmonary metastases were largely unchanged (Figure 2b). Radiology revealed a PR, with the large central tumor decreasing 51% from 9.7 × 8.8 cm to 7.4 × 5.7 cm in the axial plane, and the dorsal metastasis of the right lobe of the liver decreasing 66% from 3.1 × 2.7 cm to 1.9 × 1.5 cm in the axial plane. Pemigatinib dose was reduced to 9 mg QD to manage toxicity, but after one cycle, dose was re-escalated to 13.5 mg QD because of rising CA19-9 concentrations. The patient received three more cycles of pemigatinib at 13.5 mg QD until disease progression (Figure 2c).

Within 1 week of pemigatinib treatment, the patient experienced hyperphosphatemia, initially managed by low phosphate diet in consultation with a dietitian due to patient’s food allergies. Following unsuccessful dietary management, the phosphate binder sevelamer was introduced at 800 mg three times daily and then reduced to 800 mg twice daily to manage suspected drug-induced nausea. Hyperphosphatemia manage-ment was further complicated by widely varying blood phosphate concentrations, monitored weekly. The patient never developed hypophosphatemia; however, sevelamer was pre-emptively paused when phosphate concentrations dropped rapidly leading into off-treatment week. The patient reported dysgeusia, which did not require intervention. Stomatitis and xerostomia also occurred during the first three cycles of pemigatinib, managed with topical saline rinsing and, in part, triggered the dose reduction. Nail deformation/thickening and mild palmar skin dryness occurred after five cycles and did not require intervention.

Following disease progression on pemigatinib, reduced-dose oxaliplatin and 5-fluorouracil were initiated and then discontinued shortly thereafter due to rapid deterioration of physical condition. More extensive genomic testing was planned but not performed due to insufficient remaining biopsy sample, and the decision was made to discontinue systemic therapy. Nine months after diagnosis, the patient was moved to palliative care and died approximately 1 month later.

### Case 2: Patient history, diagnosis, disease progression, and pemigatinib treatment

**Figure 3:**
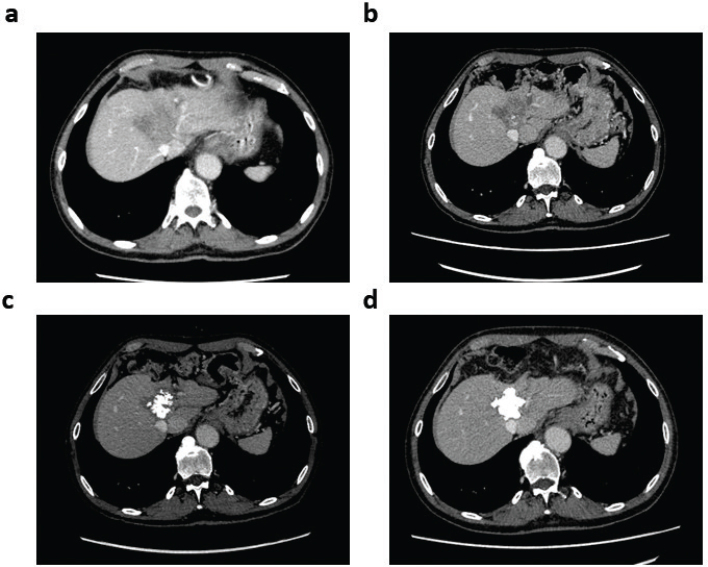
Tumor calcification in the patient with CCA on long-term pemigatinib treatment. Axial CT images show (a) stable disease after 13 cycles of GemCis chemotherapy, (b) 20% reduction in tumor size after three cycles of pemigatinib, (c) increasing tumor calcification after six cycles of pemigatinib, and (d) complete tumor calcification after approximately 3.5 years on pemigatinib. CCA: cholangiocarcinoma; CT: computed tomography; GemCis: gemcitabine + cisplatin.

The patient was an 81-year-old man at diagnosis of locally advanced, unresectable iCCA. Except for known asymptomatic aortic stenosis, he was healthy before iCCA diagnosis. He presented with acute upper right abdominal pain after a meal, initially suspected as gallstones. Abdominal ultrasound followed by CT scan revealed a large intrahepatic tumor; proficient mismatch repair iCCA was confirmed by biopsy. Pathologically enlarged lymph nodes were observed. Upon CCA confirmation, the patient received nine cycles of first-line cisplatin and gemcitabine, followed by four cycles of gemcitabine monotherapy over 9 months (Figure 1b). The patient’s best response to standard-of-care therapy was stable disease (Figure 3a).

Approximately 3 months after diagnosis, archival diagnostic biopsy tissue was subjected to molecular profiling with the Oncomine Comprehensive Assay v3 NGS and RNA panels (ThermoFisher Scientific, Waltham, MA, USA). An *FGFR2-BICC1* fusion was detected. Chemotherapy was discontinued, and pemigatinib 13.5 mg QD was initiated on an intermittent dosing schedule (2 weeks on, 1 week off per labeled indication). At cycle 9, the dosing schedule was maintained, but pemigatinib was reduced to 9 mg QD to ameliorate adverse events (AEs). After 2 years of treatment, the dosing schedule was changed, based on shared decision-making, to a 2-weeks-on, 2-weeks-off schedule, and pemigatinib was maintained at 9 mg QD. Response to pemigatinib was evident on CT scan three cycles after treatment initiation, with a 20% reduction in tumor size (Figure 3b). After six cycles, intratumoral calcifications were visible on CT scan (Figure 3c). The latest scan showed complete tumor calcification (Figure 3d), which appears to be a surrogate of treatment effect.

AEs experienced while receiving pemigatinib include mild hyperphosphatemia (controlled by diet without need for phosphate binders), mild epistaxis and mucositis, grade 2 nail toxicity (resolved on-treatment), alopecia with hair regrowth, mild fatigue, mild myalgia, and arthralgia. Most AEs resolved after dose reduction. At the time of writing, the patient has received ongoing pemigatinib for 48 months. The patient has an Eastern Cooperative Oncology Group performance status of 0, is fully active and is able to perform strenuous work, including manual labor.

## Discussion

As precision medicine in CCA (especially iCCA) is rapidly evolving, it is increasingly important to have abundant initial biopsy tissue for all potential further analyses in addition to initial diagnosis, such as NGS, which is recommended for CCA [9]. As CCA is a very aggressive disease, relevant predictive tests should be performed early during first-line treatment to minimize delay in initiating second-line therapy.

Intratumoral calcification induced by FGFR inhibition as observed in the second patient with extraordinary response has been reported, to our knowledge, only once previously. Calcification of a phosphaturic mesenchymal tumor harboring an *FN1-FGFR1* fusion was observed in a patient with tumor-induced osteomalacia treated with FGFR inhibitor infigratinib; however, the patient ultimately experienced disease progression and died [10]. Based on the experience of our patient described here, calcification of iCCA tumors could be considered a surrogate for treatment response as has been observed for some, but not all, cancers [11, 12].

The cases described here highlight the importance of managing AEs associated with pemigatinib. Both patients experienced hyperphosphatemia, which was not surprising based on the safety profile reported in the FIGHT-202 clinical trial. In that trial, hyperphosphatemia (59%), alopecia (50%), and diarrhea (48%) were the most frequently observed TEAEs; however, they did not lead any patients to discontinue pemigatinib [8]. Several TEAEs observed in these two patients were also observed in FIGHT-202: fatigue (44%), stomatitis (38%), dysgeusia (36%), skin dryness (22%), epistaxis (14%), and myalgia (12%) [8]. The most common grade ≥3 TEAEs in FIGHT-202 were hypophosphatemia (14.3%), stomatitis (6.8%), and arthralgia (6.1%) [8]. Hyperphosphatemia is generally managed through dietary intervention or phosphate-lowering therapy [13]; however, patient-specific factors such as food allergies may complicate dietary management. Physicians should consider how hyperphosphatemia management changes during off-treatment weeks, such as discontinuing low-phosphate diets or phosphate-binders to avoid hypophos-phatemia. Medications may be prescribed at the beginning of treatment for most common AEs: loperamide for diarrhea and metoclopramide for nausea. Consider the role of other healthcare providers, such as nurses specialized in administering and managing pemigatinib treatment, who can assist in counseling patients on management of mild AEs, including hyperphosphatemia. If eye-related AEs develop, patients should be referred to an ophthalmologist immediately. Further, routine blood testing and monitoring is necessary.

Both European Society for Medical Oncology and National Comprehensive Cancer Network guidelines advocate for a dual approach that includes both tissue biopsies and DNA analyses to optimize patient management, monitor disease progression, and identify resistance mechanisms [14, 15]. Active research suggests that patients with certain mutations upon disease progression may benefit from treatment with alternative *FGFR2* inhibitors; however, these findings require confirmation in large randomized clinical trials [14, 16].

These cases also illustrate the importance of patient and caregiver education and physician interaction. Patients and caregivers should be counseled to contact their oncologist promptly instead of waiting until the next scheduled visit to raise concerns or report AEs. For example, the pemigatinib regimen was changed to a 2-weeks-on, 2-weeks-off schedule at the request of the second patient. This illustrates how pemigatinib therapy can be modified to meet individual patient needs, possibly improving treatment adherence and prolonging therapy duration.

In conclusion, our cases demonstrate variability of pemigatinib treatment responses in two patients from Nordic countries and underscore the importance of AE management and patient education to optimize management.

## Author contributions

Both authors were responsible for the conception, design, reporting, writing, and approval of this article.

## Data Availability

Access to individual patient-level data is not available for these case studies. Information on Incyte’s data sharing policy and instructions for submitting data requests are available at: https://www.incyte.com/Portals/0/Assets/Compliance%20and%20Transparency/clinical-trial-data-sharing.pdf?ver=2020-05-21-132838-960 Further inquiries can be directed to datasharing@incyte.com.
